# Supplementary material for: Slow conformational exchange and overall rocking motion in ubiquitin protein crystals
Source: Nat Commun. 2017 Jul 27;8:145. doi: 10.1038/s41467-017-00165-8 (PMC5529581; doi:10.1038/s41467-017-00165-8)
Supplement: Supplementary file 1 — Supplementary Information [file 41467_2017_165_MOESM1_ESM.pdf]

File name: Supplementary Information
Description: Supplementary figures, supplementary table, supplementary methods and supplementary references.

File name: Supplementary Movie 1
Description: Excerpt from 2-µs MD trajectory of cubic-PEG-ub crystal of ubiquitin. The animation shows two ubiquitin molecules extracted from the simulated crystal unit cell: molecule #26 representing chain A and molecule #11 representing the proximal chain B (colored gold and blue, respectively). The sites $K11_{chainA}$, $D52_{chainB}$ and $G53_{chainB}$ are shown using a stick representation. The movie covers the time interval from 350 to 625 µs; the coordinates of chain A at 350 ns are included as a reference structure (pale gold) to highlight the rocking motion of this molecule. Early in the movie one can observe the formation of $K11_{chainA}$-$D52_{chainB}$ salt bridge accompanied by the $D52_{chainB}$/$G53_{chainB}$ peptide plane flip, which corresponds to the βI → βII transition. After $K11_{chainA}$-$D52_{chainB}$ salt bridge is established, $K11_{chainA}$ also engages $D52_{chainB}$(CO) (which is generally a rare interaction).

File name: Supplementary Movie 2
Description: Excerpt from 2-µs MD trajectory of MPD-ub crystal of ubiquitin. The animation shows single ubiquitin molecule extracted from the simulated cell (which is comprised of four crystal unit cells). The main chain of the β turn residues 51-54 is shown using a stick representation. The movie covers the time interval from 700 to 1000 ns; at the time point 823 ns the system undergoes transition from βII to βI conformation, followed by formation of the signature R54-E51 hydrogen bond. Also included in the movie are: *(i)* the animated graph of the torsional angle φ(t) in the residue G53, which is indicative of the βI / βII status; *(ii)* the animated graph of the amide 15N chemical shift δ(t) for residue R54, with average δ values in the βI and βII forms indicated by horizontal dashed lines; *(iii)* experimental relaxation dispersion data for residue R54 in the MPD-ub crystal fitted using the fast exchange model (continuous lines). The motional time scale according to the experimental data, 69 µs, is one-to-two orders of magnitude slower than suggested by the MD simulations (the same is true for other manifestations of µs dynamics in ubiquitin crystals).

File name: Supplementary Movie 3
Description: Excerpt from 2-µs MD trajectory of cubic-PEG-ub crystal of ubiquitin. The animation shows three ubiquitin molecules extracted from the simulated crystal unit cell: molecule #28 (chain A, colored blue), #39 (chain B, colored gold), and #14 (chain A, colored green). Residues H68, Q40, and E64 belonging to the three respective molecules are shown using stick representation. The movie covers the time interval from 800 to 2000 ns. The side chain of residue H68 is initially in χ1=-60° conformation and makes intermolecular hydrogen bond with the side chain of residue Q40. At the time point 888 ns it undergoes a transition to χ1=180° conformation, establishing hydrogen bond with the side chain of residue E64, and then reverts to its initial state at the time point 1714 ns. Other details can be found in the caption of Figure 6. It is also appropriate to comment here on the algorithms used to calculate ^{15}N chemical shifts. Our calculations using SHIFTX program produced the average chemical shift difference Δδ=2.9 ppm for the two rotameric states of H68 (corresponding to the gap between the two horizontal dashed lines shown in the plot). Alternative calculations using SHIFTX+ module from the program SHIFTX2 resulted in a qualitatively similar picture, but yielded significantly smaller Δδ=1.0 ppm. Finally, SHIFTX+ calculations including intermolecular environment produced very similar result, Δδ=0.9 ppm. We conclude that predicted Δδ values are loaded with substantial uncertainty, which reflects the intrinsic limitations of the chemical shift predictors. Given this level of uncertainty, it is unnecessary to include the intermolecular environment in the chemical shift calculations since it produces only minor changes in the calculated ^{15}N shifts.

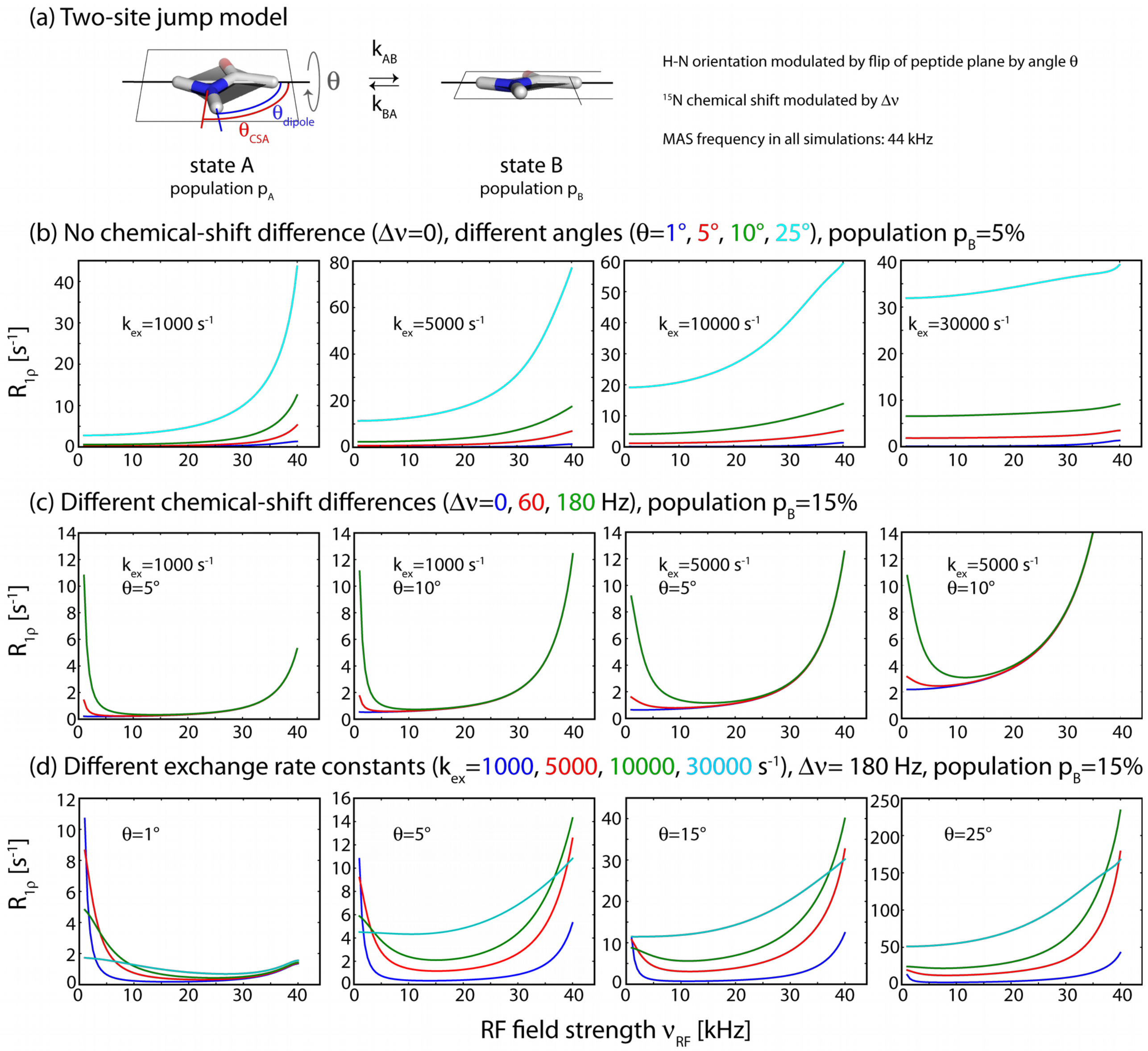

Supplementary Figure 1: Properties of $R_{1\rho}$ relaxation dispersion experiments under MAS revealed by numerical simulations using the program GAMMA.[1] The evolution of ^{15}N transverse magnetization in a two-spin ^{1}H-^{15}N spin system was monitored over 150 ms (a typical relaxation delay used in our experiments), and fitted mono-exponentially to extract a decay rate constant which is plotted as a function of the RF field strength. (a) Graphical representation of the exchange model. Jump of the peptide plane was assumed corresponding to rotation about Cα-Cα axis by an angle θ, by an angle ϕ, at a rate constant $k_{ex}=k_{AB}+k_{BA}$, involving the two states populated at levels of p_A and p_B, respectively. We assumed that the NH bond is at an angle of 83°, and the principal axis of the axially symmetric ^{15}N CSA tensor at 103° to the rotation axis. The NH dipolar coupling was set to 10.6 kHz and the ^{15}N CSA anisotropy was Δσ=157 ppm (at 600 MHz). These values assume a fast-motion (not inducing $R_{1\rho}$ decay) with an order parameter of S=0.92 (S^2=0.85) and standard values for a HN bond length of 1.02 Å and a CSA anisotropy of -170 ppm. Panels (b)-(d) show RD curves for different combinations of exchange rate constants, jump angles, chemical-shift differences and populations. Note that the two effects that result from conformational dynamics, i.e. isotropic chemical-shift modulation and angular modulation of dipolar coupling (as well as CSA) are additive, and give rise to non-flat dispersions in different regions of the RD curve. In other words, both BMCRD and NERRD effects make appearance in the simulated relaxation dispersion profiles.

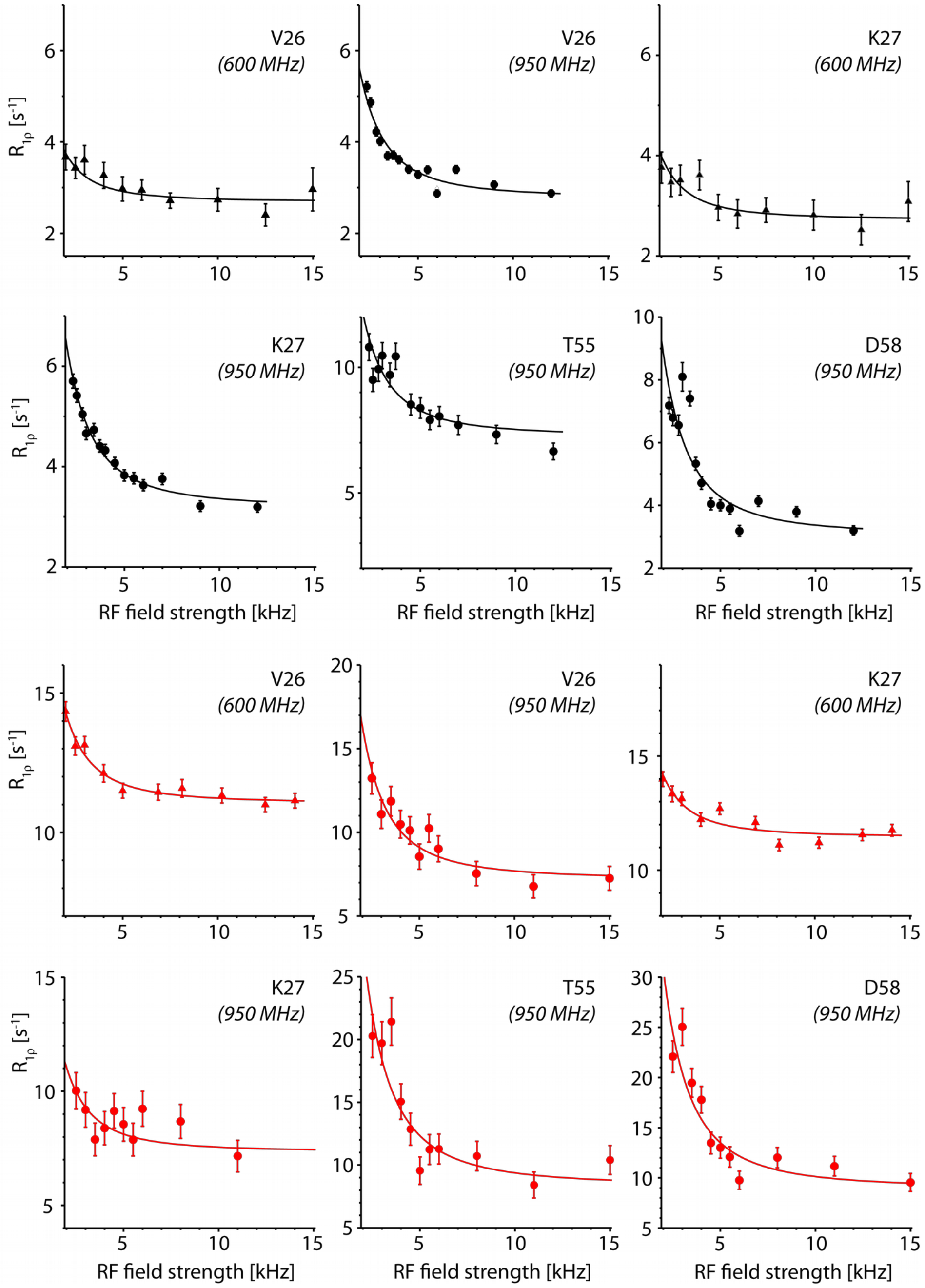

Supplementary Figure 2: BMCRD data of the cluster of 5 residues which were used for the BMCRD fit reported in Figure 1. A total of 9 BMCRD profiles were used in the fit of each of the crystal forms, i.e. the three BMCRD profiles shown in Figure 1 and the six BMCRD profiles shown here.

(A)

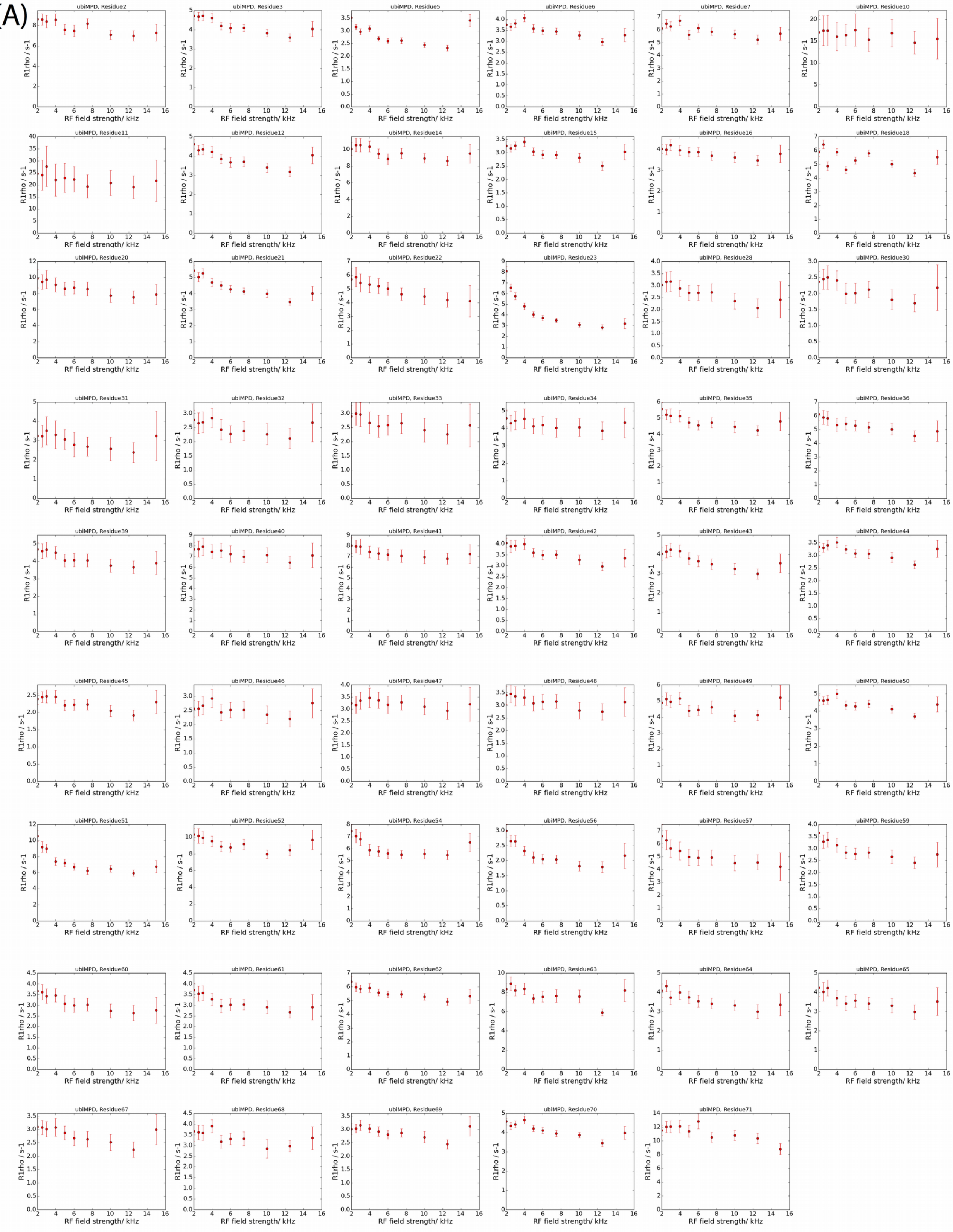
ubiMPD, Residue2
ubiMPD, Residue3
ubiMPD, Residue5
ubiMPD, Residue6
ubiMPD, Residue7
ubiMPD, Residue10
ubiMPD, Residue11
ubiMPD, Residue12
ubiMPD, Residue14
ubiMPD, Residue15
ubiMPD, Residue16
ubiMPD, Residue18
ubiMPD, Residue20
ubiMPD, Residue21
ubiMPD, Residue22
ubiMPD, Residue23
ubiMPD, Residue28
ubiMPD, Residue30
ubiMPD, Residue31
ubiMPD, Residue32
ubiMPD, Residue33
ubiMPD, Residue34
ubiMPD, Residue35
ubiMPD, Residue36
ubiMPD, Residue39
ubiMPD, Residue40
ubiMPD, Residue41
ubiMPD, Residue42
ubiMPD, Residue43
ubiMPD, Residue44
ubiMPD, Residue45
ubiMPD, Residue46
ubiMPD, Residue47
ubiMPD, Residue48
ubiMPD, Residue49
ubiMPD, Residue50
ubiMPD, Residue51
ubiMPD, Residue52
ubiMPD, Residue54
ubiMPD, Residue56
ubiMPD, Residue57
ubiMPD, Residue59
ubiMPD, Residue60
ubiMPD, Residue61
ubiMPD, Residue62
ubiMPD, Residue63
ubiMPD, Residue64
ubiMPD, Residue65
ubiMPD, Residue67
ubiMPD, Residue68
ubiMPD, Residue69
ubiMPD, Residue70
ubiMPD, Residue71
R1rho / s-1
RF field strength/ kHz

(B)

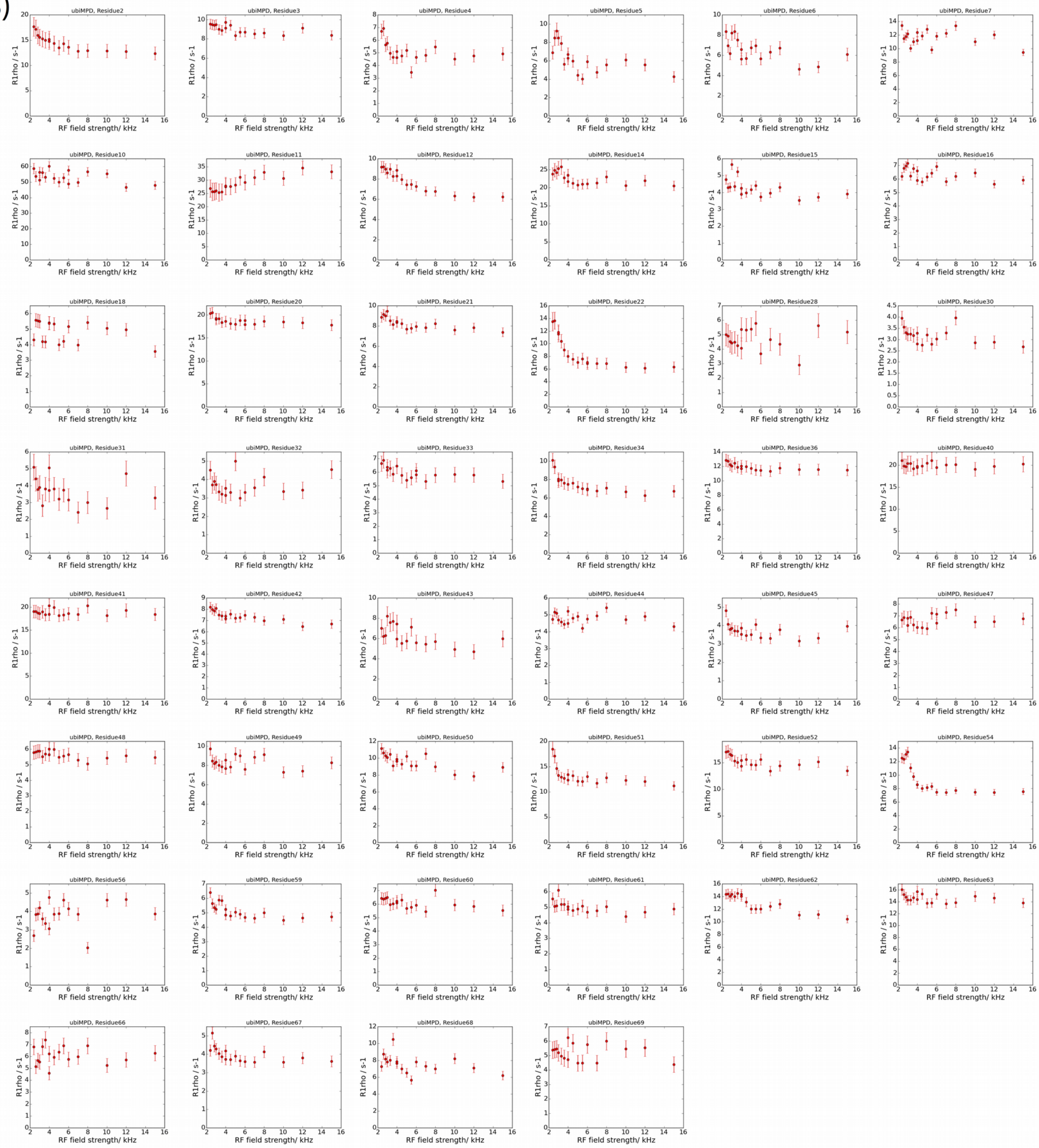
ubiMPD, Residue2
ubiMPD, Residue3
ubiMPD, Residue4
ubiMPD, Residue5
ubiMPD, Residue6
ubiMPD, Residue7
ubiMPD, Residue10
ubiMPD, Residue11
ubiMPD, Residue12
ubiMPD, Residue14
ubiMPD, Residue15
ubiMPD, Residue16
ubiMPD, Residue18
ubiMPD, Residue20
ubiMPD, Residue21
ubiMPD, Residue22
ubiMPD, Residue28
ubiMPD, Residue30
ubiMPD, Residue31
ubiMPD, Residue32
ubiMPD, Residue33
ubiMPD, Residue34
ubiMPD, Residue36
ubiMPD, Residue40
ubiMPD, Residue41
ubiMPD, Residue42
ubiMPD, Residue43
ubiMPD, Residue44
ubiMPD, Residue45
ubiMPD, Residue47
ubiMPD, Residue48
ubiMPD, Residue49
ubiMPD, Residue50
ubiMPD, Residue51
ubiMPD, Residue52
ubiMPD, Residue54
ubiMPD, Residue56
ubiMPD, Residue59
ubiMPD, Residue60
ubiMPD, Residue61
ubiMPD, Residue62
ubiMPD, Residue63
ubiMPD, Residue66
ubiMPD, Residue67
ubiMPD, Residue68
ubiMPD, Residue69
R1rho / s-1
RF field strength/ kHz

(C)

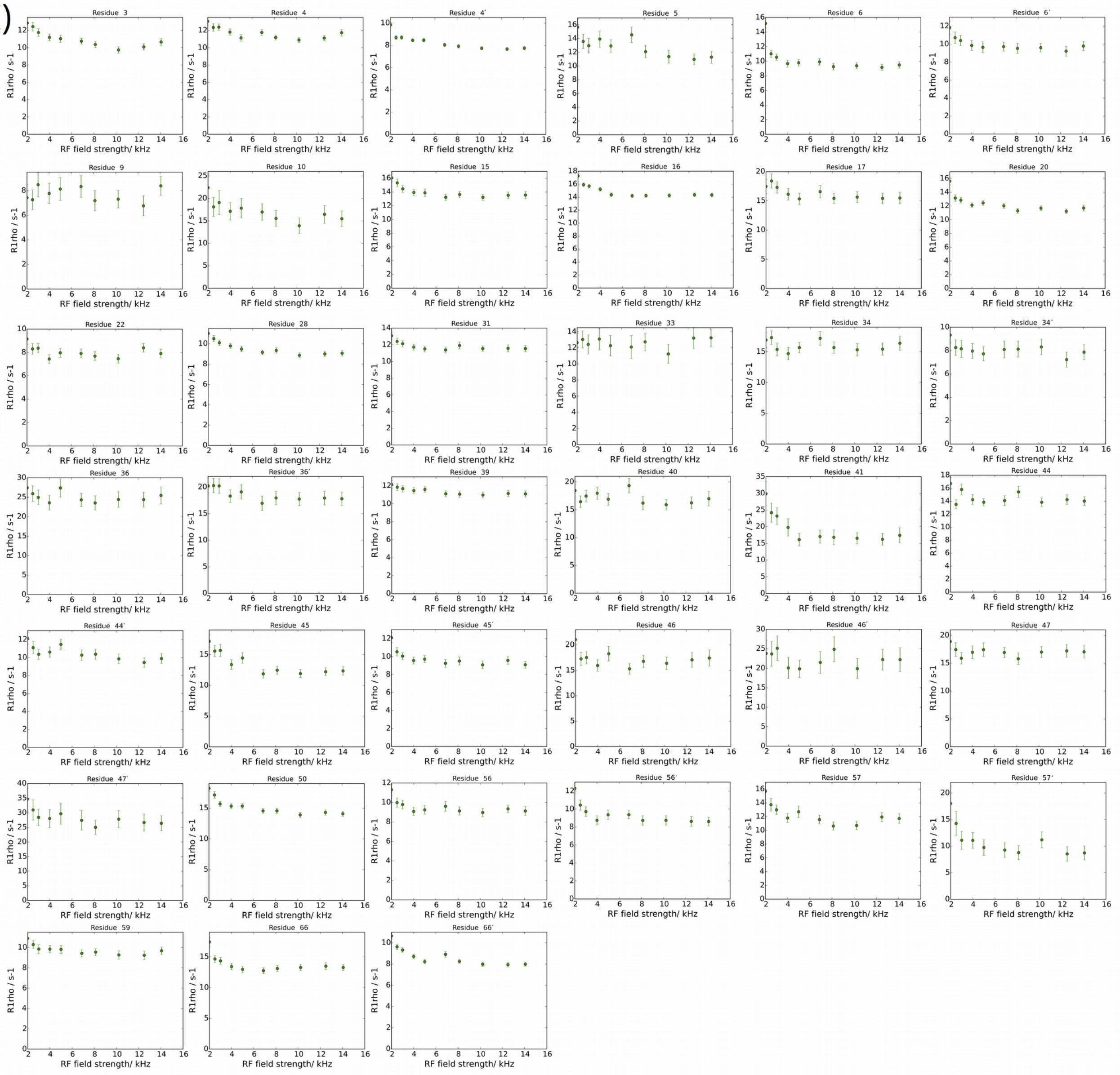

Residue 3
Residue 4
Residue 4´
Residue 5
Residue 6
Residue 6´
Residue 9
Residue 10
Residue 15
Residue 16
Residue 17
Residue 20
Residue 22
Residue 28
Residue 31
Residue 33
Residue 34
Residue 34´
Residue 36
Residue 36´
Residue 39
Residue 40
Residue 41
Residue 44
Residue 44´
Residue 45
Residue 45´
Residue 46
Residue 46´
Residue 47
Residue 47´
Residue 50
Residue 56
Residue 56´
Residue 57
Residue 57´
Residue 59
Residue 66
Residue 66´
R1rho / s-1
RF field strength/ kHz

(D)

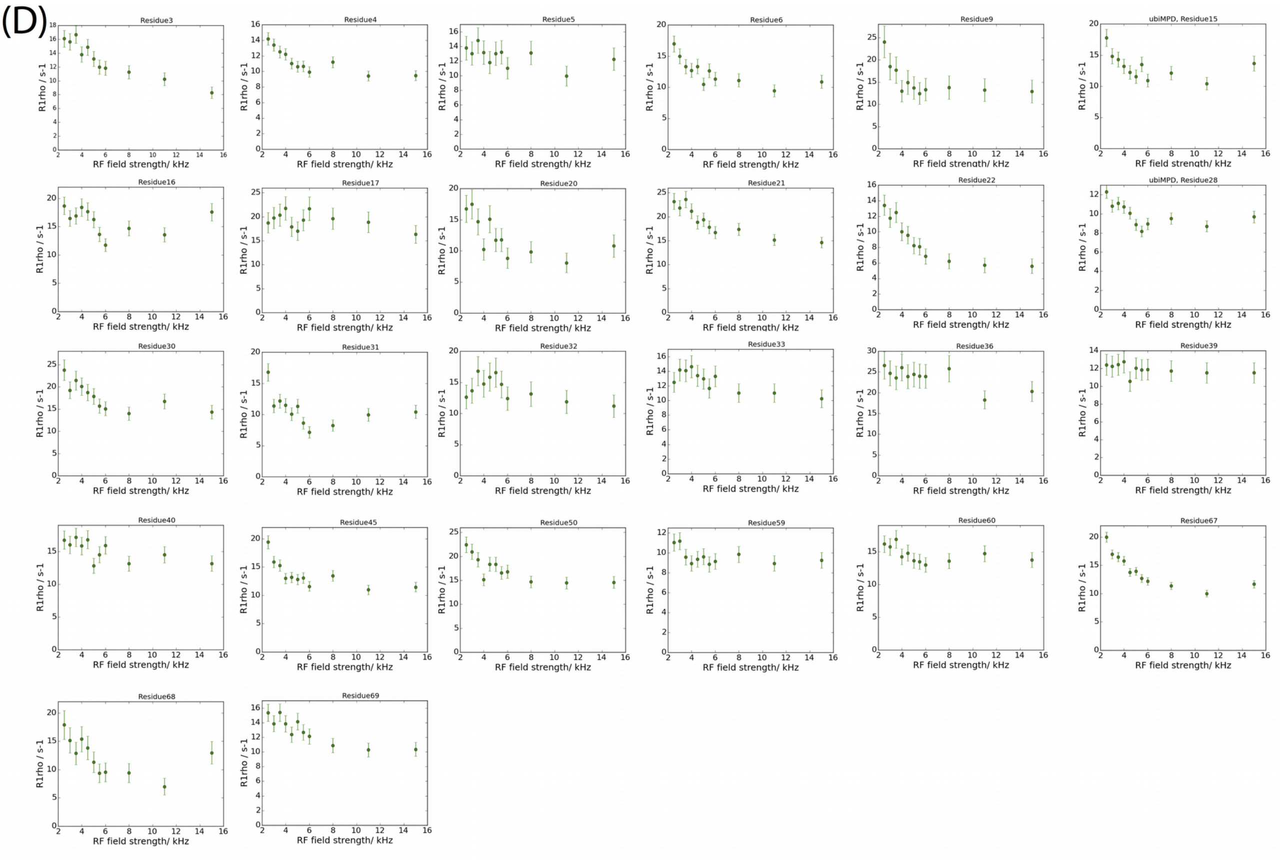

Supplementary Figure 3. BMCRD data of residues in MPD-ub crystals (a,b) and cubic-PEG-ub crystals (c,d) at 600 MHz (a,c) and 950 MHz (b,d) ^{1}H Larmor frequency.

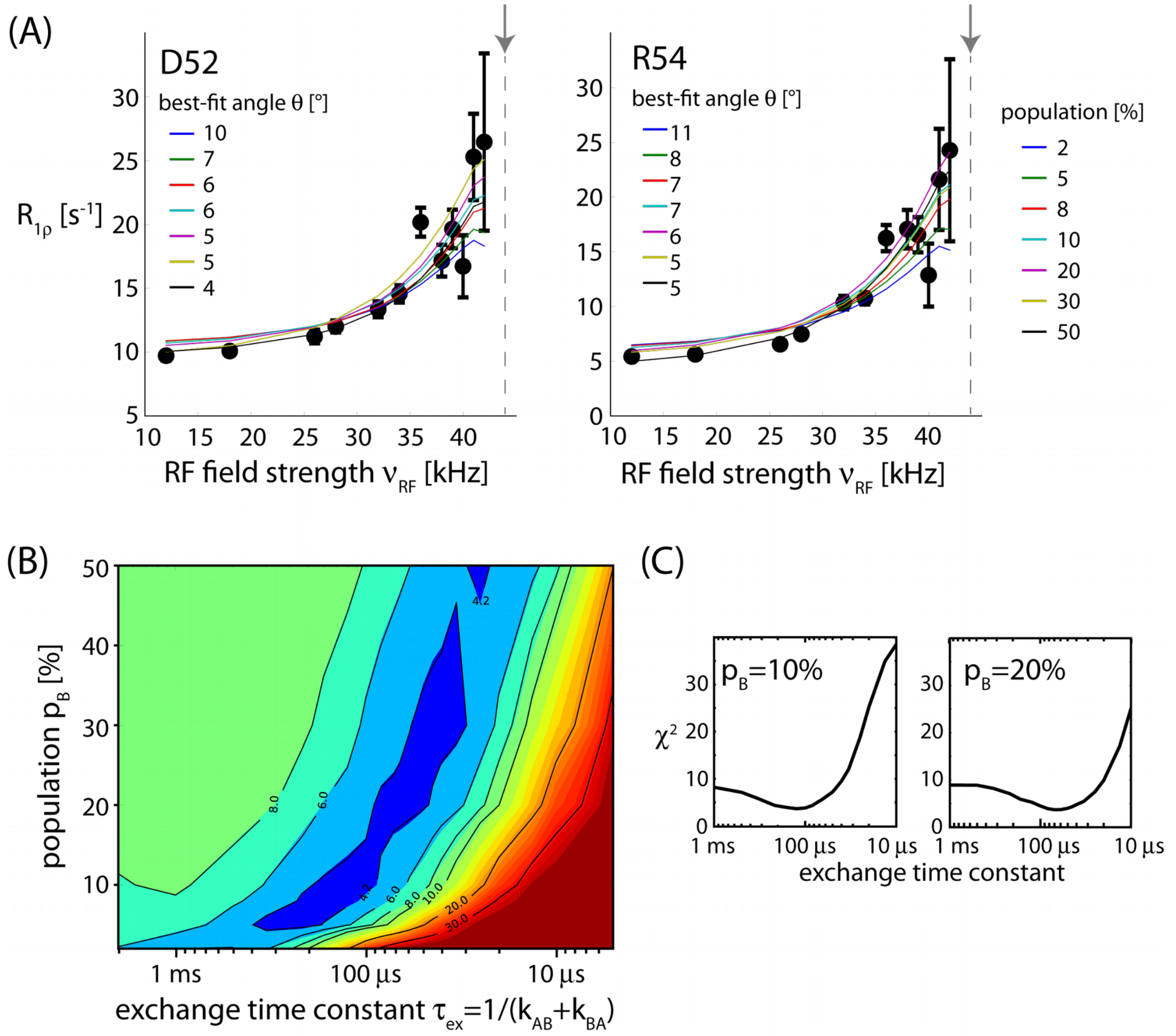

Supplementary Figure 4. (A) NERRD data of D52 and R54 in MPD-ub crystals, obtained at a MAS frequency of 44.053 kHz. To fit these data, a set of numerical simulations of the ^{15}N relaxation was performed, as described before,[2] assuming a two-site jump model. Diffusive motion models cannot be implemented within our simulation framework, but it has been shown that two-site jump models can be related to more general model-free-like models.[3] In this grid search the following parameters were varied: population, time scale of exchange, angle of jump. The data of these two residues were jointly fitted to a single motional process, in which populations and exchange time constant are common, while the angle is fitted individually to each residue. In addition, RF-field independent plateau value was also fitted for each profile. This plateau $R_{1\rho}$ value stems from fast dynamics, which are not represented in our simulations. The NERRD profiles simulated in this manner were compared to the experimental profiles and the *rms* deviation χ^2 was calculated. Panel (B) shows the χ^2 for this grid-search procedure as a function of the exchange time constant and the population of the involved states (with jump angles and plateau values optimized at each individual point on the grid). Panel (C) shows traces of χ^2 surface at two selected minor-state population p_B. Panel (A) in addition to the experimental data also shows a series of simulated NERRD profiles obtained with different combinations of jump angles θ and minor-state population p_B (color-coded as indicated in the plot).

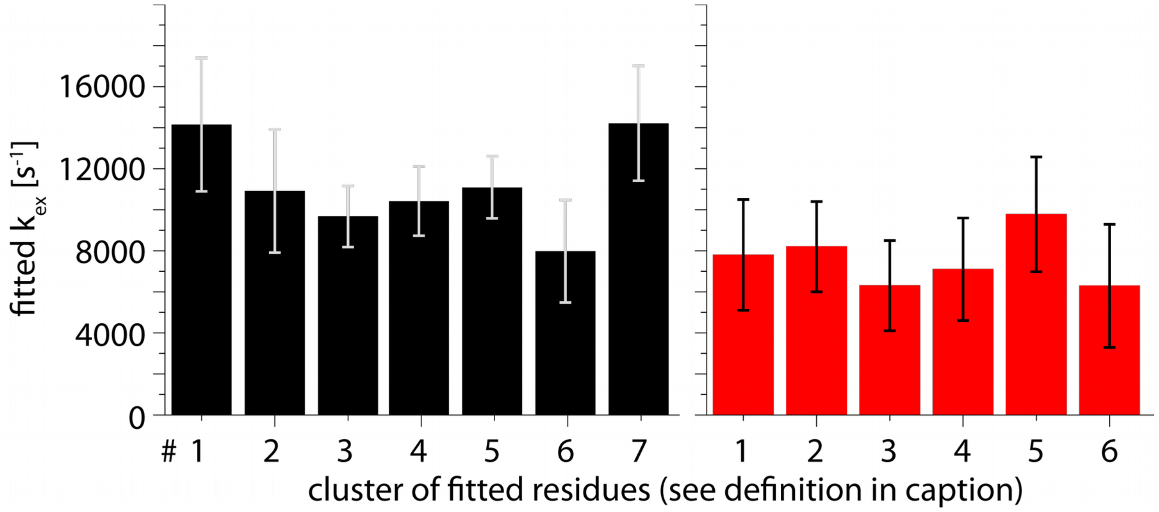

Supplementary Figure 5. Exchange rate constants from fits of a two-state exchange model to BMCRD data of MPD-ub (black) and cubic-PEG-ub (red), using data from different residues and different B_0 field strengths. These data show that the exact outcome of the fit does not significantly depend on the choice of the subset of data. The data used in these fits are (residue numbers at field strength): **MPD-ub: 1**: 22, 23, 26, 27, 54, 55, 56, 57, 58 at 600 MHz; **2**: 22, 23, 26, 54, 55, 56, 57, 58 at 950 MHz; **3**: 26, 27, 55, 58 at 600, 950 MHz; **4**: 55, 58 at 600, 950 MHz and 23 at 950 MHz; **5**: 27, 55, 58 at 600, 950 MHz and 23 at 950 MHz; **6**: 26, 27, 55, 58 at 600 MHz and 23 at 950 MHz; **7**: 23, 26, 27, 55, 58 at 600 MHz; **cubic-PEG-ub: 1**: 23, 26, 27, 55, 58 at 950 MHz; **2**: 26, 27, 55, 58 at 600 MHz and 23 at 950 MHz; **3**: 55, 58 at 600, 950 MHz and 23 at 950 MHz; **4**: 27, 55, 58 at 600, 950 MHz and 23 at 950 MHz; **5**: 26, 27, 50, 55, 58 at 600 MHz; **6**: 20, 55, 58, 59 at 600, 950 MHz.

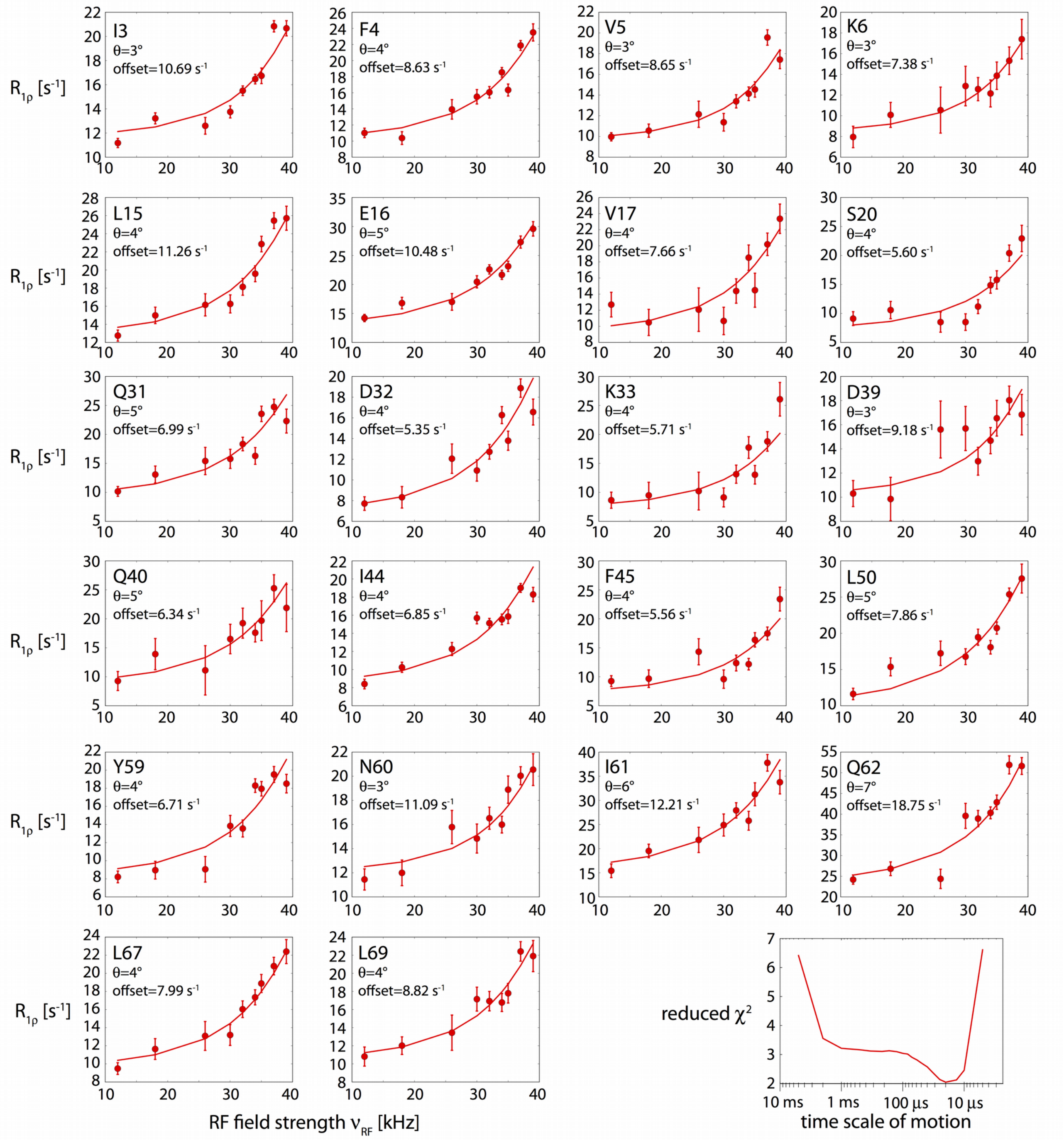

Supplementary Figure 6. NERRD profiles measured in cubic-PEG-ub, at a MAS frequency of 44.053 kHz. For this selection of 22 residues we have excluded those residues that are in the β-turn and the adjacent region (22-27 and 51-58), and data with low signal/noise or resonance overlap. The data of all these residues were fitted against numerical spin-dynamics simulations (see description of NERRD fits below). The solid lines show the best-fit curves, using the globally optimized exchange time constant of 20 μs and residue-wise individually optimized jump angles. In performing these fits, a constant $R_{1\rho}$ offset (i.e. plateau value) was additionally fitted to the data set of each residue. The best-fit values of the jump angles and the offsets are indicated in the panels. The lower right panel shows the global reduced χ^2 value plotted over the grid of exchange time constants.

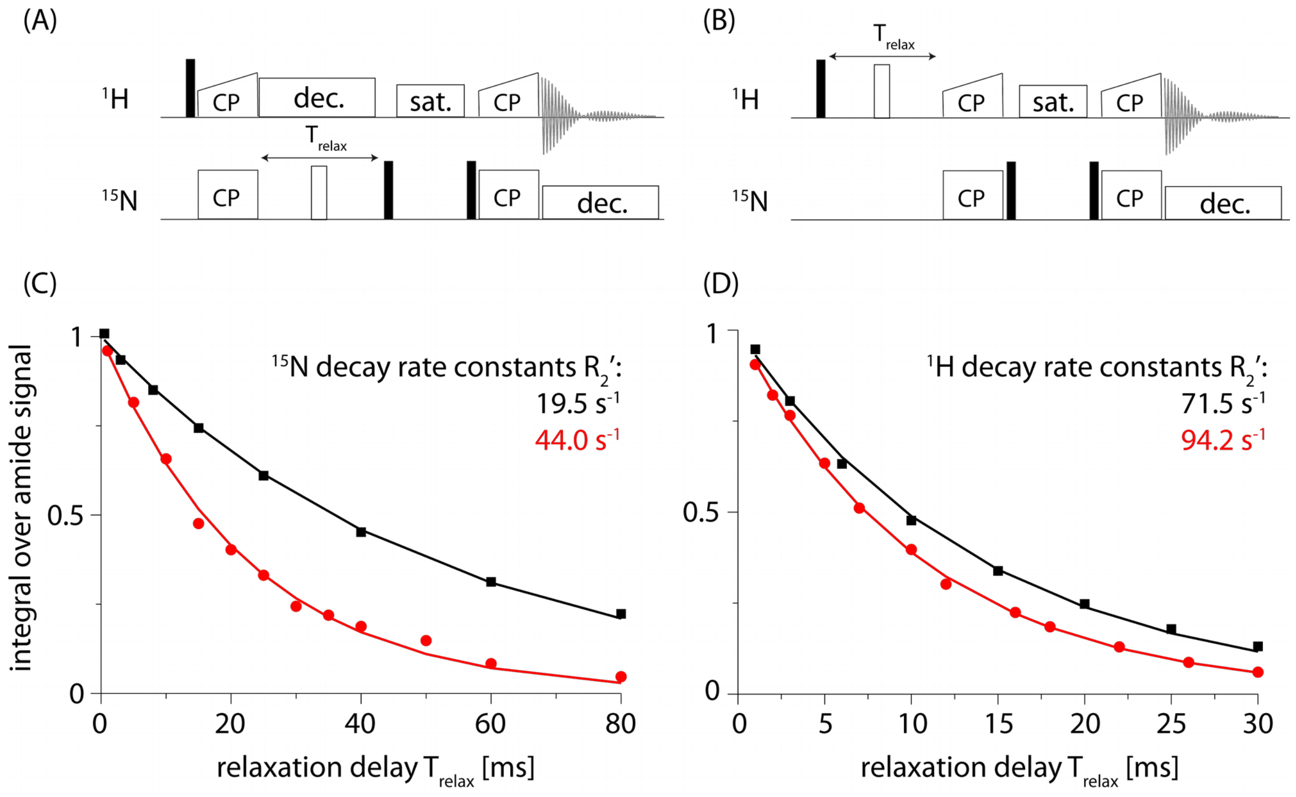

Supplementary Figure 7. Transverse relaxation differs in the two crystal forms, MPD-ub and cubic-PEG-ub. (A,B) Pulse sequences used to measure the effective transverse decay rate constnat, R_2', of ^{15}N (A) and ^{1}H (B) spins, performed as 1D measurements, providing an estimate over the entire amide signal. In (A) a 10 kHz WALTZ-16 decoupling is used on ^{1}H spins during the ^{15}N relaxation delay. (C,D) Experimental data of MPD-ub (black) and cubic-PEG-ub (red) samples, obtained from integrating the amide band (6.9 to 9.5 ppm) in a series of 1D spectra with different delays T_{relax}. The samples were uniformly ^{2}H,^{15}N,^{13}C-labeled, and prepared in 80% D_2O/20% H_2O, as described in the Methods section. Measurements were performed at a MAS frequency of 53 kHz, and an effective sample temperature of 300 K. Mono-exponential fits are shown as solid lines and the corresponding decay rate constants are indicated. These data show that even though the structures of ubiquitin in the two crystal forms are very similar and the labeling scheme is identical, spin coherences in cubic-PEG-ub decay faster, pointing to an important role of dynamics in determining ssNMR line widths in these crystals.

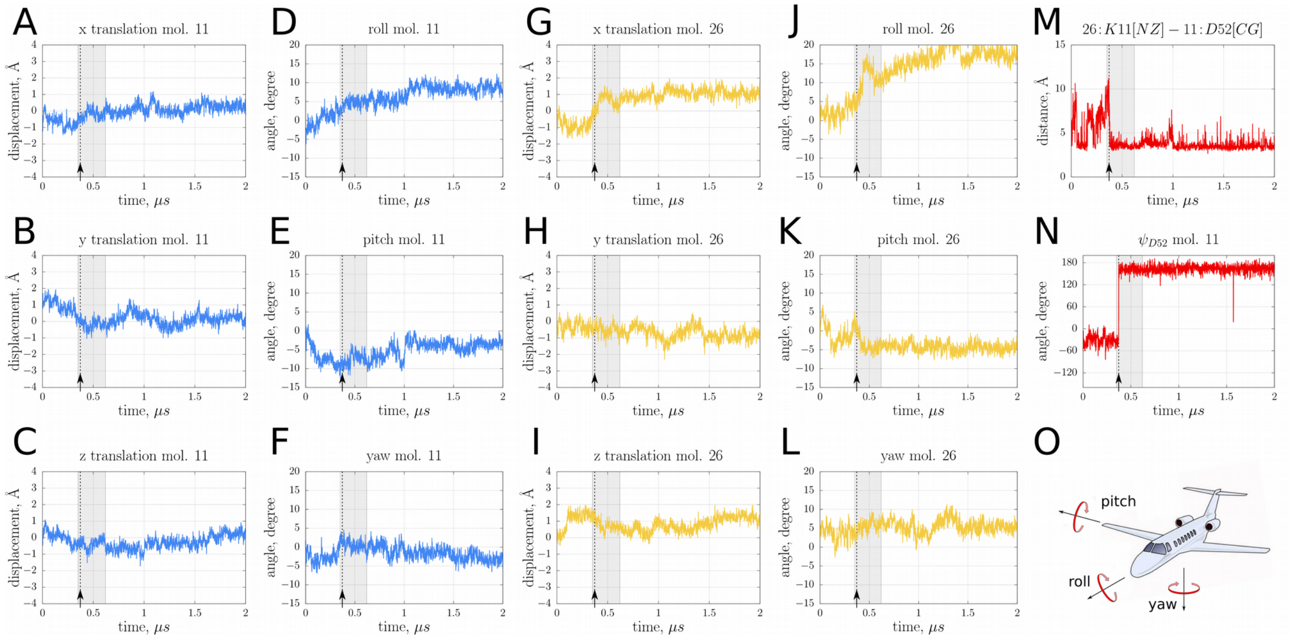

Supplementary Figure 8. (A-L) Overall motion of the two ubiquitin molecules from the MD trajectory of cubic-PEG-ub crystal. The selected ubiquitin molecules are the same as shown in Movie S1, with graphs colored accordingly: gold color for molecule #26 (chain A), blue color for molecule #11 (chain B). Shown are the displacements of the center of mass of the two ubiquitin molecules relative to their position in the original crystal 3N30, as well as the variables describing the reorientational motion of the molecules (roll, pitch, and yaw). Grey band in the plots indicate the duration of the Supplementary Movie 1. The arrow indicates the moment when the salt bridge $K11_{chainA}$-$D52_{chainB}$ was formed and the βI → βII transition occurred. (M) Distance between $K11_{chainA}$ Nζ and $D52_{chainB}$ Cγ which is indicative of the salt bridge formation. (N) Torsional angle ψ in residue $D52_{chainB}$ indicative of the βI/βII conformation. (O) Scheme illustrating the definition of roll, pitch, and yaw. Prior to the calculations, the MD data were preprocessed to (*i*) undo the effect of protein molecule jumps due to periodic boundary conditions and (*ii*) remove the motion of the overall center of mass of all protein molecules in the simulation. To parameterize rotational motions, protein molecules from each individual MD snapshot were superimposed onto their counterparts in the reference structure (via Cα atoms within helices and beta sheets); when superimposing the pair of molecules, a rotation matrix Ξ has been determined. Matrices Ξ were used to extract roll, pitch, and yaw angles; roll, pitch and yaw axes are laboratory frame axes. The observed roll of the molecule #26 (panel J) corresponds to somewhat extreme scenario – only several molecules out of 48 become so significantly reoriented during the MD simulation. Such exaggerated rocking dynamics represent the shortcomings of the MD model as discussed by us previously: the crystal essentially begins to melt during the simulation (cf. the notion of structural drift).[4]

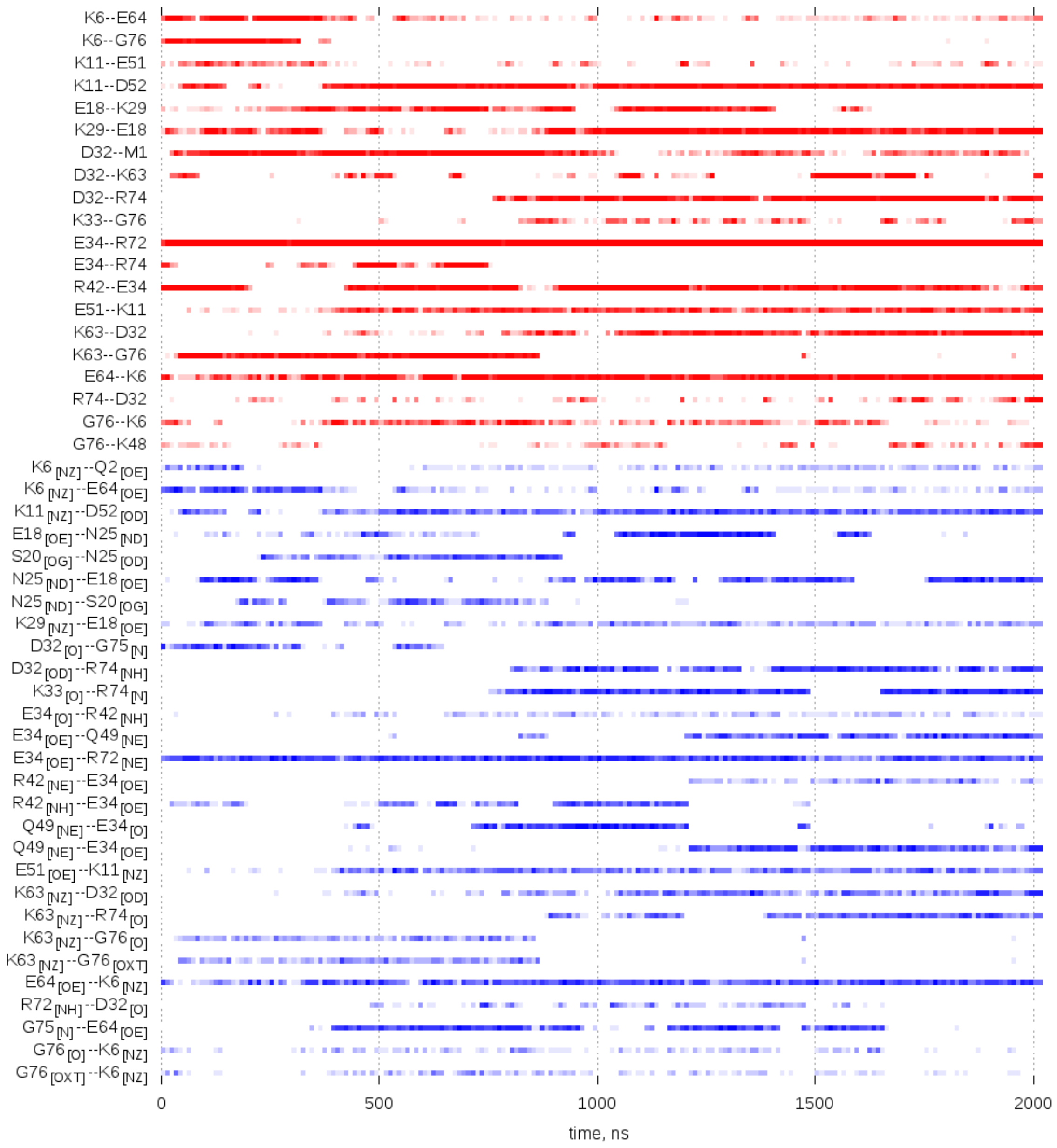

Supplementary Figure 9. Time evolution of intermolecular salt bridges (red) and intermolecular hydrogen bonds (blue) originating at the molecule #26 in 2-μs MD trajectory of cubic-PEG-ub crystal. To generate this plot the trajectory was sampled with 1-ns step and then divided into 20-ns bins. The intensity of color reflects the content of the interaction in a given bin (bright color corresponds to the persistent interaction, pale color corresponds to a sporadic interaction). Molecule #26 is the same molecule as shown in Movie S1 (painted gold).

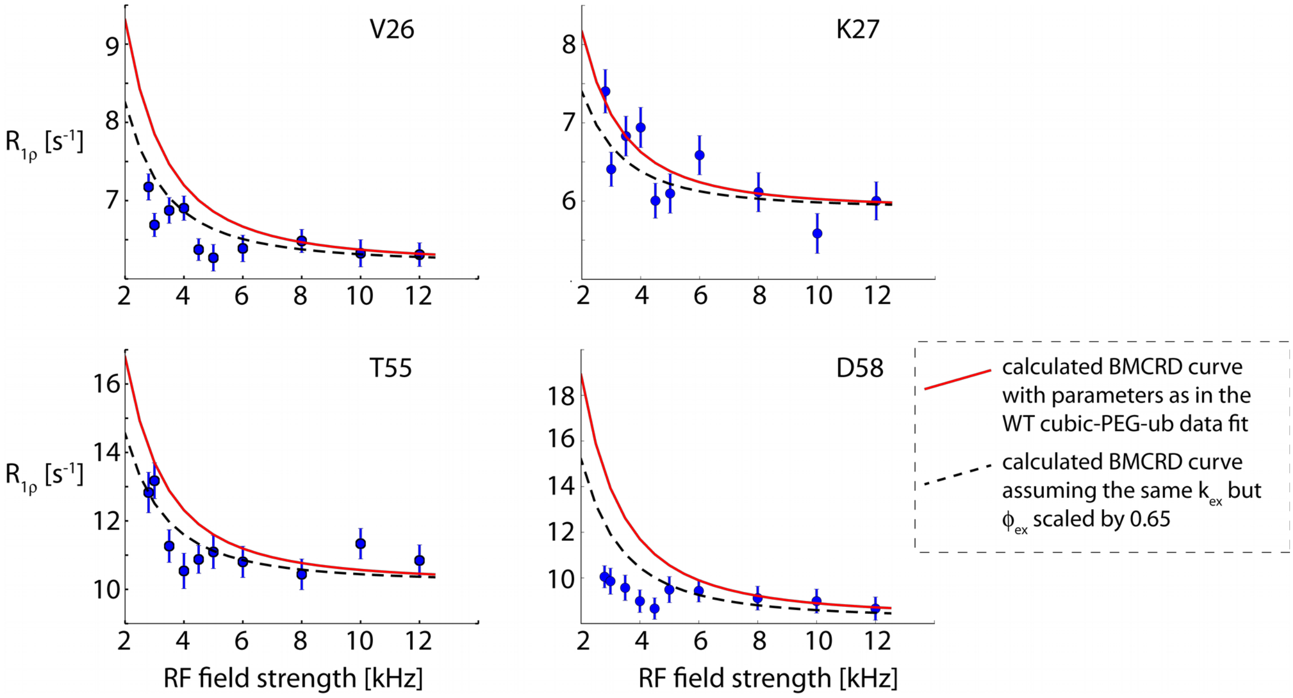

Supplementary Figure 10. Bloch-McConnell relaxation dispersion data of K11A ubiquitin in the cubic-PEG-ub crystal form, collected at a field strength of 14.1 T (600 MHz ^{1}H Larmor frequency). The sample was prepared with 20% back-protonation of exchangeable sites, and the experiment was performed at an effective sample temperature of ca. 300 K (±2 K), at a MAS frequency of 44 kHz. A series of relaxation delays up to 70 ms was employed for each RF field strength (6 data points), and the collected data were fit mono-exponentially, like for the WT samples. The figure shows the data for four out of the five residues used in the cluster fit illustrated in Figure 1; residue I23 is not resolved in 2D spectra, and could only be obtained in 3D spectra at 950 MHz used for Figure 1. Red lines shown along with the experimental data depict calculated BMCRD curves using the exchange data obtained for wild-type cubic-PEG-ub (τ_{ex}=125 μs, ϕ_{ex} as shown in Figure 1e). As an illustrative example of the effect of a change of population levels, the dashed black lines show calculated curves assuming that the residue-wise ϕ_{ex} values are reduced by a factor 0.65, compared to those shown in Figure 1e. This reduction corresponds to a drop of minor-state population from 26% (average between chains A and B according to the MD data, see Table 1) to 15%. In plotting these curves, the plateau $R_{1\rho}$ was adjusted to produce good agreement with the data points at highest RF field strength. If the mutation of Lys 11 to Ala indeed results in a reduction of the minor-state population, then the difference between red and black lines in the plot illustrates the corresponding reduction in the BMCRD amplitude. For K27 it is not clear whether such a reduction is observed. For the three other residues, however, it is evident that the experimental data indeed point to a reduced BMCRD amplitude. Although not a direct proof, these data corroborate the observation by MD simulations that mutation of K11 affects the conformational exchange equilibrium in the neighboring molecule by lowering the amount of minor state.

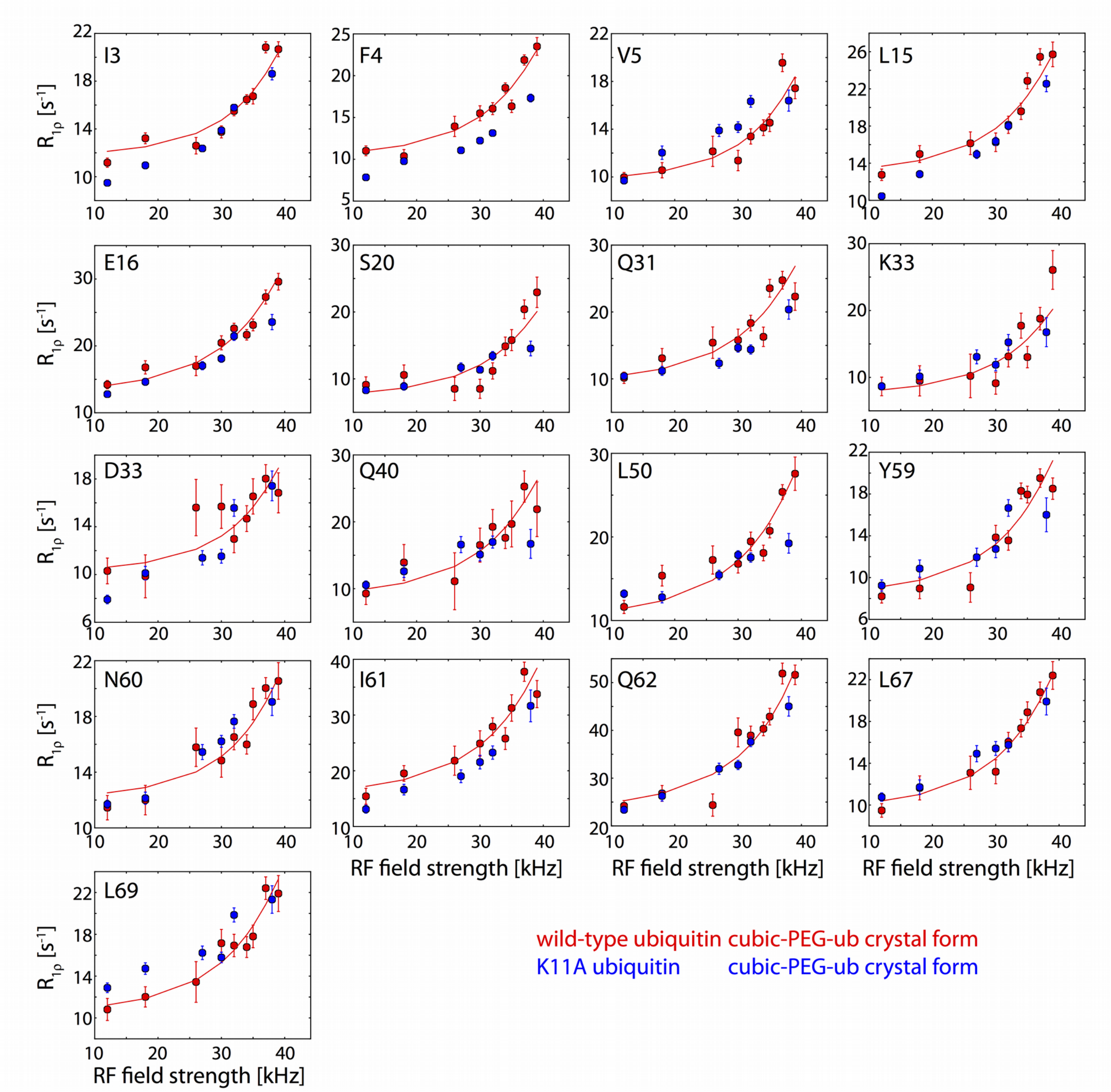

Supplementary Figure 11. NEar-Rotary-resonance Relaxation-Dispersion (NERRD) data of cubic-PEG-ub mutant K11A (blue), and comparison to equivalent data, recorded under identical conditions, of wild-type cubic-PEG-ub (red). The data were recorded at 44.053 kHz MAS frequency; see description of Figure S5 for details. The solid lines correspond to numerical fits of a 2-site exchange model with the best-fit time constant of 20 μs, as described in the caption of Figure S6. These data do not show any significant difference between WT and K11A in terms of overall rocking motion.

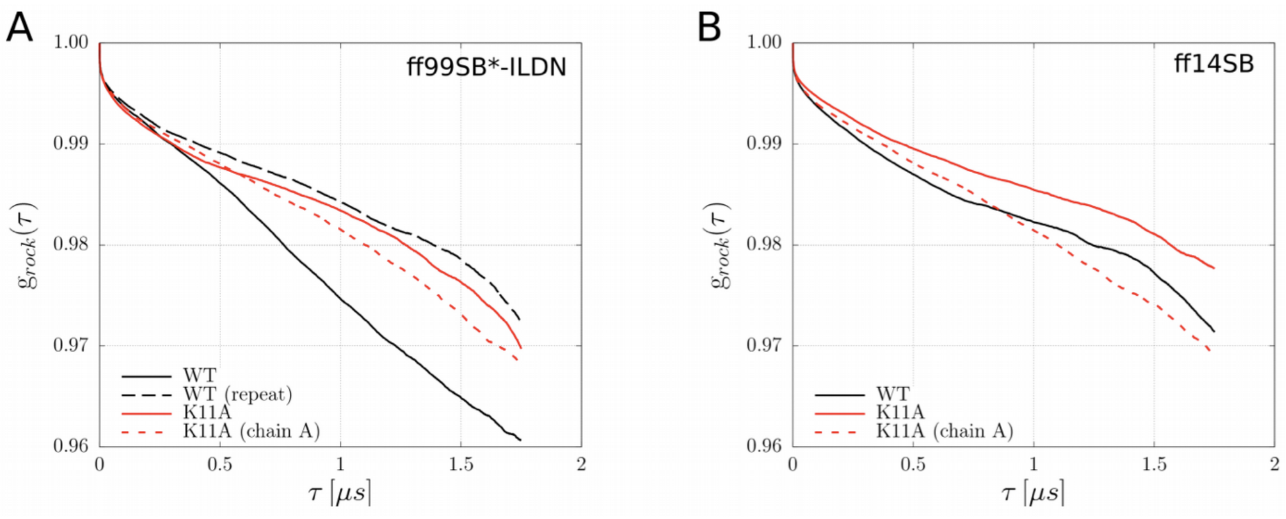

Supplementary Figure 12. Orientational rigid-body correlation functions representative of the rocking motion in the trajectories of cubic-PEG-ub crystals of wild-type ubiquitin, as well as K11A and K11A (chain A only) variants. In each case, $g_{rock}(\tau)$ have been calculated as described previously[4] and averaged over all 48 individual molecules in the trajectory, including chains A and B. The results in panel (A) are from the 2-μs simulations recorded under Amber ff99SB*-ILDN force field, while the results in panel (B) are from the equivalent simulations employing Amber ff14SB. The $g_{rock}(\tau)$ are plotted to 1.75 μs because the results toward τ = 2 μs are statistically inadequate. The data, including repeat trajectories of wild-type ubiquitin crystals, demonstrate poor convergence with respect to the amplitude of rocking. This effect can be linked to the drift of the crystal simulations, i.e. subtle "melting" of the crystal lattice during the course of the simulations. Nevertheless, all trajectories consistently demonstrate the drop in βII conformation in the K11A crystal compared to the wild-type crystals. In the case of ff99SB simulations, the proportion is 11% (K11A chain A) and 13% (K11A) vs. 26% (original wt) and 15% (duplicate wt). In the case of ff14SB simulations the proportion is 8% (K11A chain A) and 10% (K11A) vs. 16% (wt). These predictions are supported by the experimental data shown in Figure S10. Similarly, G53A simulations (not shown) demonstrate a clear shift in the βI : βII balance, which has been in part verified experimentally[5], but no sustainable evidence of altered rocking dynamics.

Assumed minor-state population in cubic-PEG-ub	*Calculated minor-state population in MPD-ub*
50 %	5 to 12 %
40 %	4.5 to 11.5 %
30 %	4 to 10 %
20 %	3 to 7 %

Supplementary Table 1. Estimation of the minor-state population in MPD-ub. These calculations are based on the assumption that the difference of the ϕ_{ex} values in cubic-PEG-ub and MPD-ub mainly reflects the different βI and βII populations in the two crystals. The experimentally determined ratio is ϕ_{ex}(cubic-PEG-ub)/ϕ_{ex}(MPD-ub) = 3.3 ±1.0, see main text. Assuming that the frequency shifts Δν between βI and βII conformations are on average the same in the two crystal forms, we thus arrive at the relationship $p_{\beta I}p_{\beta II}$(cubic-PEG-ub)/$p_{\beta I}p_{\beta II}$ (MPD-ub) = 3.3 ±1.0, which is then used to calculate the entries in the right column of the table (the range of calculated populations in MPD-ub are based on the error bar of the ϕ_{ex} ratio, and represents the range within one standard deviation).

Supplementary methods

Description of NERRD fits

We have noted before that for the case of microsecond dynamics the relaxation rate constants calculated from equation 1 do not perfectly match relaxation rate constants that are found by fitting numerical simulations of spin-relaxation with a mono-exponential decay.[6] This finding has also been reported by Smith et al.[7] The reason for this discrepancy is the fact that equation 1 correctly describes the *initial slope* of the relaxation profile.[8] In MAS ssNMR relaxation decays are inherently multi-exponential, as described in detail elsewhere.[6] If experimental data are fitted by a mono-exponential function the corresponding relaxation rate constant therefore becomes dependent on the sampling of the decay curve and does not exactly match the value calculated from equation 1.

To compensate for this discrepancy, we have adopted in this work a procedure whereby we use numerical simulations instead of Eq. 1. Briefly the evolution of the ^{15}N transverse magnetization in a 2-spin ^{1}H-^{15}N system undergoing two-site exchange is simulated by a stochastic Liouville approach, described and used elsewhere.[6,9,10] In doing so we assumed that the effect of CSA/dipolar cross-correlated relaxation is eliminated by applying a ^{1}H π pulse in the center of the relaxation period, in line with our experimental scheme.[10] We simulated the decay of ^{15}N coherence over a time scale that corresponds to the experimentally chosen maximum relaxation delay, and fitted the simulated data by an exponential function. This numerically fitted rate constant is thus directly comparable to the rate constant obtained experimentally. Such simulations were performed for all the experimentally chosen RF field strengths and MAS frequencies over a grid of dynamic parameters: exchange rate constant, population of the minor state, angle between the orientations of ^{1}H- ^{15}N dipolar vector in the two conformational states and angle between the orientations of the long axis of the axially symmetric ^{15}N CSA tensor in the two conformational states (the two angles are assumed to be identical).

The experimental $R_{1\varrho}$ RD profiles were then compared to the collection of simulated NERRD profiles to retrieve the best-fit set of dynamic parameters; hereby, a single additive fit parameter was allowed when comparing experimental and simulated NERRD curves to account for $R_{1\varrho}$ plateau in the experimental data. This plateau reflects the presence of other motional modes, such as nanosecond motions, which contribute to the experimental data, but are not considered in the simulation. These faster dynamics would not give rise to an RF-field dependent profile of $R_{1\varrho}$ rate constants, but would rather appear as an RF-field independent offset.

A χ^2 value for each simulated NERRD curve compared to the experimental NERRD data was obtained by summing the deviations $[(R_{1\varrho}^{exp} - R_{1\varrho}^{sim})/\sigma^{exp}]^2$ over all RD points; the residuals from

several residues were subsequently combined (D52 and R54 in the case of MPD-ub; 22 residues for cubic-PEG-ub). This chi-square value was further normalized by the number of degrees of freedom, to obtain a reduced χ^2, which is the value reported in Figures 2c,d, 3d, S4 and S6.

We have also investigated the possibility to jointly fit BMCRD and NERRD data points. Using a set of noise-free in silico data we found that such extended fits are similar to fits using separately only the low-RF-field ("BMCRD") or high-RF-field ("NERRD") data, and that the inclusion of NERRD data does not overcome the inherent inability to disentangle populations from $\Delta\nu$ that BMCRD fits suffer from.

MD simulations protocol

Starting coordinates for the MPD-ub trajectory were obtained from the high-resolution crystallographic structure 3ONS.[11] This structure misses four flexible C-terminal residues, which give rise to weak and uninterpretable electron density. To address this issue, we prepared 200 structural models based on 3ONS geometry, where the terminal segments were initially generated in a form of random coil[12] and then grafted onto the body of the protein (specifically, C^{α} and C' atoms in residue R72 were used as the points of attachment). Each of these models also included the crystallographic water as found in 3ONS. The resulting constructs were packed into a unit cell (space group $P3_221$, six protein molecules per unit cell) using the appropriate tool in Amber 11.[13] The original dimensions of the cell, a=b=48.41 Å and c=61.97 Å, were all multiplied by a factor 1.016 to account for thermal expansion of the protein crystal upon transition from 100 K (temperature at which 3ONS was solved) to 301 K (temperature at which ssNMR data were taken).[14]

As a next step, the protein coordinates were protonated. To determine the protonation status of individual Asp and Glu residues, we performed the PROPKA[15] calculations for ubiquitin in a crystal-lattice environment. The results were generally consistent with the estimations using solution pK_a,[16] except for several residues experiencing the effect of crystal contacts. Since charged side chains are oftentimes involved in crystal contacts, we believe that it is more appropriate to use the computed pK_a values which explicitly take into consideration the effects of crystal packing. The effective pH was assumed to be 4.2, same as in the crystallization buffer.[11] The system was then neutralized by adding eight Cl^- ions per ubiquitin molecule (forty-eight Cl^- ions per unit cell). The number of water molecules to be added to the crystal unit cell was initially estimated based on the simple density considerations.[17] This number was subsequently adjusted such as to ensure that the volume of the crystal cell remains unchanged during the MD production run. Following a series of iterative corrections, we found that it was necessary to add ca. 1650 water molecules (on top of 546 crystallographic waters already contained in the crystal unit cell). Both chlorine ions and water molecules were added using AddToBox facility[18] in Amber 11. We used the SPC/E water model,[19] which has been recommended as the preferred choice for Amber ff99SB force field;[18] this model also showed the best results in our trial simulations. No attempt was made to include 2-methyl-2,4-pentanediol, glycerol, or sodium citrate, which were also a part of the crystallization buffer.[11] None

of these compounds appear in the crystallographic structure 3ONS and it is unclear to what degree they are partitioned into the crystal. In solution, ubiquitin does not show any significant interaction with MPD.[20]

Additional manipulations were performed to optimize the coordinates of the C-terminal residues in each of the 200 starting models. To emulate the crystal lattice environment, periodic boundary conditions have been applied at the faces of the unit cell. Heavy protein atoms, except those in the four C-terminal residues, were restrained to their original coordinates (force constant 500 kcal mol^{-1} $Å^{-2}$). The system was then energy-minimized via 500 steps of steepest descent, followed by 500 steps of conjugate gradient minimization. The minimization was conducted in Amber 11 under control of Amber ff99SB force field with Best and ILDN corrections (ff99SB*-ILDN).[21–23] Subsequently, the system was heated from 0 to 1000 K and then cooled back to 0 K. In doing so, the temperature was incremented (decremented) with the step of 200 K; total duration of the heating and cooling stages was 40 and 120 ps, respectively. During this stage the heavy atoms were restrained with the force constant 10 kcal mol^{-1} $Å^{-2}$.

The 200 structural models processed according to the above scheme were subsequently ranked by energy. Toward this goal, we stripped the system of water and instead applied the implicit solvent (option igb=5 in Amber).[24] Since Amber does not allow for use of periodic boundary conditions in conjunction with implicit solvent, we have modeled the effect of crystal lattice by assembling a block of three identical unit cells. The resulting construct was once again subjected to the energy minimization, where all heavy atoms were fixed while the protons were optimized. Finally, the energy of the obtained system was evaluated using Amber ff99SB*-ILDN potential with igb=5 solvation. The results were used to rank the 200 models by energy and select 10 lowest-energy models. Next we return to the optimized models containing explicit solvent, focusing on the subset of 10 models identified in the previous step. Recall that these models essentially reproduce the unit crystal cell as seen in the crystallographic coordinate set 3ONS, but with the addition of the ubiquitin C-terminal tail. We have investigated these models and concluded that the tail moves sufficiently freely and samples the entire conformational phase space available to it in the time frame of 100 ns.[25] Therefore we have chosen one single model (the one with the lowest energy) as a starting point for all of the following simulations.

The MPD-ub simulations involved the block of four unit cells (dimensions a and b doubled). The starting coordinates were obtained by assembling multiple copies of the unit crystal cell. The system was subjected to two final rounds of energy minimization prior to the beginning of the production run. At first, water coordinates were optimized while protein atoms were fixed; then all restraints were lifted and the entire model was minimized. After that the temperature of the system was raised from 0 to 301 K by running 20 ps constant-volume simulation with weak restraints applied to all protein atoms (10 kcal mol^{-1} $Å^{-2}$). Finally, the production run was initiated. The first 20 ns of each trajectory were treated as equilibration stage and subsequently discarded. The MD simulation was run at constant pressure (1 atm) and constant temperature (301 K) using the Langevin thermostat with collision frequency 3 ps^{-1}. The constant pressure was maintained using the isotropic scaling option, with pressure relaxation time set to 2 ps. Long-range electrostatics were treated with the particle mesh Ewald method;[26] non-bonded Coulomb and Lennard-Jones

interactions were truncated at 9 Å. The bonds involving hydrogen atoms were constrained using SHAKE algorithm. The integration step was 2 fs and the protein coordinates were stored every 5 ps.

The periodic boundary contained 24 ubiquitin molecules and 8,772 water molecules, for the total of 56,244 atoms. The volume of the simulation box remains stable throughout the simulation within 0.5% of its target value (on average, there is a slight uniform expansion as described by linear factor 1.0009). The production rate with NVIDIA GeForce GTX580 cards was 9 ns per card per day. The net length of the trajectory was 2 μs.

The same approach was employed to record the cubic-PEG-ub trajectory. In this case the initial coordinates were derived from the crystallographic structure 3N30.[27] The periodic boundary box was modeled after a single crystal unit cell, containing 48 ubiquitin molecules (equally divided between chains A and B) and 23,419 water molecules. The volume of the simulation box remains stable throughout the simulation within 0.7% of its target value (on average, there is a slight uniform contraction as described by linear factor 0.9986). Note that the statistical sampling for both chain A and chain B is the same as for the single ubiquitin chain in the MPD-ub trajectory. The repeat trajectory of cubic-PEG-ub was recorded using Amber 16[28] equipped with Amber ff99SB*-ILDN force field. The production rate using two NVIDIA GeForce GTX1080 cards was 38 ns per day.

The solution trajectory was based on the coordinate file 1UBQ;[29] this crystal structure has an excellent record in terms of interpreting the solution NMR data. The sample conditions were assumed to be pH 4.7, 300 K, matching those in the experimental study.[30] The truncated octahedral periodic boundary box contained a single ubiquitin molecule and 3,572 water molecules. The net length of the solution trajectory was 10 μs.

Supplementary References

(1) Smith, S.; Levante, T.; Meier, B.; Ernst, R. Computer Simulations in Magnetic Resonance. An Object-Oriented Programming Approach. *J. Magn. Reson.* **1994**, *106*, 75–105.

(2) Ma, P.; Haller, J. D.; Zajakala, J.; Macek, P.; Sivertsen, A. C.; Willbold, D.; Boisbouvier, J.; Schanda, P. Probing Transient Conformational States of Proteins by Solid-State R1rho Relaxation-Dispersion NMR Spectroscopy. *Angew. Chem. Int. Ed.* **2014**, *53* (17), 4312–4317.

(3) Clore, G. M.; Driscoll, P. C.; Wingfield, P. T.; Gronenborn, A. M. Analysis of the Backbone Dynamics of Interleukin-1 Beta Using Two-Dimensional Inverse Detected Heteronuclear 15N-1H NMR Spectroscopy. *Biochemistry* **1990**, *29* (32), 7387–7401.

(4) Ma, P.; Xue, Y.; Coquelle, N.; Haller, J. D.; Yuwen, T.; Ayala, I.; Mikhailovskii, O.; Willbold, D.; Colletier, J.; Skrynnikov, N. R.; et al. Observing the Overall Rocking Motion of a Protein in a Crystal. *Nat. Commun.* **2015**, *6*, 8361.

(5) Gauto, D. F.; Hessel, A.; Rovó, P.; Kurauskas, V.; Linser, R.; Schanda, P. Protein Conformational Dynamics Studied by 15N and 1H R1ρ Relaxation Dispersion: Application to Wild-Type and G53A Ubiquitin Crystals. *Solid State Nucl. Magn. Reson.* **2017**, 36–44.

(6) Schanda, P.; Ernst, M. Studying Dynamics by Magic-Angle Spinning Solid-State NMR Spectroscopy: Principles and Applications to Biomolecules. *Prog. Nucl. Magn. Reson. Spectrosc.* **2016**, *96*, 1–46.

(7) Smith, A. A.; Testori, E.; Cadalbert, R.; Meier, B. H.; Ernst, M. Characterization of Fibril Dynamics on Three Timescales by Solid-State NMR. *J. Biomol. NMR* **2016**, *65* (3–4), 171–191.

(8) Torchia, D. A.; Szabo, A. Spin-Lattice Relaxation in Solids. *J. Magn. Reson.* **1982**, *49* (1), 107–121.

(9) Skrynnikov, N. Asymmetric Doublets in MAS NMR: Coherent and Incoherent Mechanisms. *Magn. Reson. Chem.* **2007**, *45*, S161–S173.

(10) Kurauskas, V.; Weber, E.; Hessel, A.; Ayala, I.; Marion, D.; Schanda, P. Cross-Correlated Relaxation of Dipolar Coupling and Chemical-Shift Anisotropy in Magic-Angle Spinning R1ρ NMR Measurements: Application to Protein Backbone Dynamics Measurements. *J. Phys. Chem. B* **2016**, *120* (34), 8905–8913.

(11) Huang, K.-Y.; Amodeo, G. A.; Tong, L.; McDermott, A. The Structure of Human Ubiquitin in 2-Methyl-2,4-Pentanediol: A New Conformational Switch. *Protein Sci.* **2011**, *20* (3), 630–639.

(12) Feldman, H. J.; Hogue, C. W. A Fast Method to Sample Real Protein Conformational Space. *Proteins* **2000**, *39* (2), 112–131.

(13) Case, D. A.; Cheatham, T. E.; Darden, T.; Gohlke, H.; Luo, R.; Merz, K. M.; Onufriev, A.; Simmerling, C.; Wang, B.; Woods, R. J. The Amber Biomolecular Simulation Programs. *J. Comput. Chem*. 2005, pp 1668–1688.

(14) Juers, D. H.; Matthews, B. W. Reversible Lattice Repacking Illustrates the Temperature Dependence of Macromolecular Interactions. *J. Mol. Biol.* **2001**, *311* (4), 851–862.

(15) Bas, D. C.; Rogers, D. M.; Jensen, J. H. Very Fast Prediction and Rationalization of pKa Values for Protein-Ligand Complexes. *Proteins* **2008**, *73* (3), 765–783.

(16) Sundd, M.; Iverson, N.; Ibarra-Molero, B.; Sanchez-Ruiz, J. M.; Robertson, A. D. Electrostatic Interactions in Ubiquitin: Stabilization of Carboxylates by Lysine Amino Groups. *Biochemistry* **2002**, *41* (24), 7586–7596.

(17) Harpaz, Y.; Gerstein, M.; Chothia, C. Volume Changes on Protein Folding. *Structure* **1994**, *2* (7), 641–649.

(18) Cerutti, D. S.; Le Trong, I.; Stenkamp, R. E.; Lybrand, T. P. Simulations of a Protein Crystal: Explicit Treatment of Crystallization Conditions Links Theory and Experiment in the Streptavidin-Biotin Complex. *Biochemistry* **2008**, *47* (46), 12065–12077.

(19) Berendsen, H. J. C.; Grigera, J. R.; Straatsma, T. P. The Missing Term in Effective Pair Potentials. *J. Phys. Chem.* **1987**, *91* (24), 6269–6271.

(20) Tollinger, M.; Sivertsen, A. C.; Meier, B. H.; Ernst, M.; Schanda, P. Site-Resolved Measurement of Microsecond-to-Millisecond Conformational-Exchange Processes in Proteins by Solid-State NMR Spectroscopy. *J. Am. Chem. Soc.* **2012**, *134* (36), 14800–14807.

(21) Hornak, V.; Abel, R.; Okur, A.; Strockbine, B.; Roitberg, A.; Simmerling, C. Comparison of Multiple Amber Force Fields and Development of Improved Protein Backbone Parameters. *Proteins* **2006**, *65* (3), 712–725.

(22) Lindorff-Larsen, K.; Maragakis, P.; Piana, S.; Eastwood, M. P.; Dror, R. O.; Shaw, D. E. Systematic Validation of Protein Force Fields against Experimental Data. *PLoS One* **2012**, *7* (2).

(23) Best, R. B.; Hummer, G. Optimized Molecular Dynamics Force Fields Applied to the Helix-Coil Transition of Polypeptides. *J. Phys. Chem. B* **2009**, *113* (26), 9004–9015.

(24) Onufriev, A.; Bashford, D.; Case, D. A. Exploring Protein Native States and Large-Scale Conformational Changes with a Modified Generalized Born Model. *Proteins* **2004**, *55* (2), 383–394.

(25) Xue, Y.; Skrynnikov, N. R. Ensemble MD Simulations Restrained via Crystallographic Data: Accurate Structure Leads to Accurate Dynamics. *Protein Sci.* **2014**, *23* (4), 488–507.

(26) Essmann, U.; Perera, L.; Berkowitz, M. L.; Darden, T.; Lee, H.; Pedersen, L. G. A Smooth Particle Mesh Ewald Method. *J. Chem. Phys.* **1995**, *103* (19), 8577–8593.

(27) Arnesano, F.; Belviso, B. D.; Caliandro, R.; Falini, G.; Fermani, S.; Natile, G.; Siliqi, D. Crystallographic Analysis of Metal-Ion Binding to Human Ubiquitin. *Chem - Eur. J.* **2011**, *17* (5), 1569–1578.

(28) Case, D. A. R.M. Betz, D.S. Cerutti, T.E. Cheatham, III, T.A. Darden, R.E. Duke, T.J. Giese, H. Gohlke, A.W. Goetz, N. Homeyer, S. Izadi, P. Janowski, J. Kaus, A. Kovalenko, T.S. Lee, S. LeGrand, P. Li, C. Lin, T. Luchko, R. Luo, B. Madej, D. Mermelstein, K.M. Merz, G. Monard, H. Nguyen, H.T. Nguyen, I. Omelyan, A. Onufriev, D.R. Roe, A. Roitberg, C. Sagui, C.L. Simmerling, W.M. Botello-Smith, J. Swails, R.C. Walker, J. Wang, R.M. Wolf, X. Wu, L. Xiao and P.A. Kollman (2016), AMBER 2016, University of California, San Francisco

(29) Vijay-Kumar, S.; Bugg, C. E.; Cook, W. J. Structure of Ubiquitin Refined at 1.8 Å Resolution. *J. Mol. Biol.* **1987**, *194* (3), 531–544.

(30) Lienin, S. F.; Bremi, T.; Brutscher, B.; Brüschweiler, R.; Ernst, R. R. Anisotropic Intramolecular Backbone Dynamics of Ubiquitin Characterized by NMR Relaxation and MD Computer Simulation. *J. Am. Chem. Soc.* **1998**, *120* (38), 9870–9879.
